# Supplementary material for: Impact of cytokine storm on severity of COVID-19 disease in a private hospital in West Jakarta prior to vaccination
Source: PLoS One. 2022 Jan 25;17(1):e0262438. doi: 10.1371/journal.pone.0262438 (PMC8789122; doi:10.1371/journal.pone.0262438)
Supplement: Attachment — Submitted filename: Response to reviewers comments PLOS ONE.docx [file pone.0262438.s001.docx]

**Rebuttal**

**Impact of Cytokine Storm on severity of COVID-19 infection in a private Hospital in West Jakarta Prior to Vaccination**

**After correction the title**

**Impact of Cytokine Storm on severity of COVID-19 disease in a private Hospital in West Jakarta Prior to Vaccination**

**For Editor**

When submitting your revision, we need you to address these additional requirements.

1. Please ensure that your manuscript meets PLOS ONE's style requirements, including those for file naming. The PLOS ONE style templates can be found at 
<https://journals.plos.org/plosone/s/file?id=wjVg/PLOSOne_formatting_sample_main_body.pdf> and 
<https://journals.plos.org/plosone/s/file?id=ba62/PLOSOne_formatting_sample_title_authors_affiliations.pdf>

Answer : We have corrected as the style requirement provided in the guideline template

2. Thank you for stating the following in the Acknowledgments Section of your manuscript: 
We would like to acknowledge the Ministry of Education and Research Indonesia (Kemenristek Dikti) and Education fund management agency (LPDP Indonesia) for their support under the World Professor Class (WPC) programme.

We note that you have provided funding information that is not currently declared in your Funding Statement. However, funding information should not appear in the Acknowledgments section or other areas of your manuscript. We will only publish funding information present in the Funding Statement section of the online submission form. 
Please remove any funding-related text from the manuscript and let us know how you would like to update your Funding Statement. Currently, your Funding Statement reads as follows: 
The author(s) received no specific funding for this work. Only the funded to held the WCP programme not for publication

Please include your amended statements within your cover letter; we will change the online submission form on your behalf.

Answer : We have added it in the cover letter. It is compulsory for us to make it in acknowledgment, but actually it is not the funding for research or publication. The funding was for conducting the World Class Professor program which led to the development of the paper.

3. We note that you have stated that you will provide repository information for your data at acceptance. Should your manuscript be accepted for publication, we will hold it until you provide the relevant accession numbers or DOIs necessary to access your data. If you wish to make changes to your Data Availability statement, please describe these changes in your cover letter and we will update your Data Availability statement to reflect the information you provide.

Answer : Please publish it after it is accepted for publication. No changes of data

4. Please include captions for your Supporting Information files at the end of your manuscript, and update any in-text citations to match accordingly. Please see our Supporting Information guidelines for more information: <http://journals.plos.org/plosone/s/supporting-information>.

Answer : We have made the changers as per the guideline

5. Please review your reference list to ensure that it is complete and correct. If you have cited papers that have been retracted, please include the rationale for doing so in the manuscript text, or remove these references and replace them with relevant current references. Any changes to the reference list should be mentioned in the rebuttal letter that accompanies your revised manuscript. If you need to cite a retracted article, indicate the article’s retracted status in the References list and also include a citation and full reference for the retraction notice.

Answer : We have revised it accordingly, thank you for the kind suggestion.

**Reviewer 1**

1. Did authors find any global data on female COVID-19 patients with cytokine storms? This will be noteworthy to know and authors can include this data to their study.

Answer : Unfortunately, to date, there is no reported global data on female COVID-19 patients with cytokine storm, to date, since our data is the first reported one on male.

2. Did authors/or any other find cytokine storms in relatively younger COVID-19 patients and how was the response to the cytokine storms?

Answer : Similar to point 1 above, since our sample population was mostly among the older age group (mean age of 60 years), we could not report on the incidence of cytokine storms in relatively younger COVID-19 patients as well as their responses. It would be interesting to see the differences in a future study, using our data as a basis for the comparison.

3. At least one supplementary Figure as illustration may be afforded as to highlight the summary or prospect of this study.

Answer : We have included a supplementary Figure accordingly. Refer to Figure 2.

4. Author may explain how to adjust the limitation in their study?

Answer : This is something that is challenging since they are indeed the limitations of the study. Nevertheless, we have attempted to explain it more clearly in the text now and have added another statement: “Additionally, since our data reported mainly on males and also the elderly, it would be interesting to see the incidence in females as well as in the younger age group and their responses against cytokine storms”. Thank you for the suggestion.

5. The abbreviations should be cross validated in the manuscript (First define them fully followed by abbreviation) and one paragraph can be added for abbreviations.

Answer: We have added it in the newly-revised manuscript. Thank you for the suggestion.

**Reviewer 2**

Reviewer #2: This retrospective study investigated the impact of cytokine storm on severity of COVID-19 disease in a private Hospital in West Jakarta Prior to Vaccination. The authors should distinguish infection from disease, because in COVID-19 disease, SARS-CoV-2 infection severity (high viral load) is not correlated to COVID-19 disease severity [1]

In Title, “COVID-19 infection” should be “COVID-19 disease”: This has been changed accordingly, thank you.

Page 5, line 5, “severity of COVID-19 infection” should be “severity of COVID-19 disease”. This has been changed accordingly, thank you.

Page 6, line 12 from bottom, “more severe infection” should be “more severe disease”. This has been changed accordingly, thank you.

Page 9, line 6, “the severity of COVID-19 infection” should be “the severity of COVID-19 disease”. This has been changed accordingly, thank you.

Page 9, Table 1, row 24, “Severity of COVID-19 infection” should be “Severity of COVID-19 . This has been changed accordingly, thank you.

disease”
Page 11, line 3 from bottom, “COVID-19 infection” should be “COVID-19 disease”. This has been changed accordingly, thank you.

Page 12, line 6, “following COVID-19 infection” should be “following SARA-CoV-2 viral infection”. This has been changed accordingly, thank you.

Page 14, last line, “patients with COVID-19 infection” should be “patients with COVID-19 disease”. This has been changed accordingly, thank you.

Page 15, line 9, “COVID-19 infection” should be “COVID-19 disease”. This has been changed accordingly, thank you.

Page 16, Conclusion, line 2, “severity of infection” should be “severity of disease”. This has been changed accordingly, thank you.

Answer: We have added all in the newly-revised manuscript. Thank you for the suggestion.

**Reviewer 3**

Reviewer #3: In this paper entitled "Impact of cytokine storm on the severity of COVID-19 infection in a private hospital in West Jakarta prior vaccination", the authors used the convenience sampling method and included subjects who are not vaccinated. The clinal outcomes of the study are fascinating. The manuscript showed that cytokine storm was more in males (mean age 60) than women and interestingly, all hypertension/diabetes subjects experienced cytokine storm. It has many exciting findings which make this manuscript engaging and lucid. However, It has minor concerns.

Thank you for the overall positive comments on our paper. We highly appreciate it.

Minor comments:

1) Please add limitations of the study.

Answer : We have added the limitation of the study in the manuscript accordingly

The study has some limitations since the duration is rather short and the fact that the published care data to date came from a small observational data (no more than 250 patients). Additionally, the types of treatment administered are not based on the WHO guideline but rather, on the local context, besides being conducted during the time when vaccines have not been introduced in the country. We have elaborated further to include the fact that future study should focus on females and the younger age group since our sample includes mostly males and the elderly.

2) The cytokine storm is a fatal reaction of human immune system. Cytokine storm is present in influenza, SARS, and MERS. In few lines, please compare it. (doi:10.1016/j.clim.2020.108652).

Thank you very much for the excellent suggestion. We have added a paragraph “Besides COVID-19, cytokine storms are also present in influenza, SARS, and MERS. Based on the immunopathology in COVID-19, SARS, and influenza infections, all the diseases show some hyperinflammation, hypercytokinemia, increased CRP levels, hyperferritinemia associated with poor prognosis, immunosuppression, lymphopenia associated with the severe disease, reduction of NK cell counts in the blood and/or NK cell dysfunction and reduction in both CD4 and CD8 T-cell counts in the blood associated with more severe disease. However, MERS tends to reveal hyperinflammation and hypercytokinemia associated with a poorer prognosis [56].” and have also quoted the said paper accordingly. Thank you.
